# Improving public health by tackling climate change

**DOI:** 10.1186/2045-4015-2-22

**Published:** 2013-06-17

**Authors:** Jenny Griffiths

**Affiliations:** 1 52 Brushfield Way, Woking, Surrey GU21 2TQ, England, UK

## Abstract

Across the world, climate change is now responsible for substantial mortality and morbidity, through direct effects on health and also by threatening the determinants of health. This commentary argues that adaptation policies to enhance resilience to adverse climate events are important, but must be coupled with determined action to reduce greenhouse gas emissions. The prize is synergy: many such policies, for example concerning food, travel and community engagement, can simultaneously improve physical and mental health.

This is a commentary on http://www.ijhpr.org/content/2/1/23.

## Commentary

On 9 May 2013, the global concentration of carbon dioxide in the atmosphere, the primary influence on recent climate change, reached 400 parts per million (ppm) for the first time in recorded history, according to data from the Mauna Loa Observatory in Hawaii. Some scientists argue that carbon dioxide must be limited to about 350 ppm in order to prevent “dangerous” climate change [1].

The World Health Organisation (WHO) estimates that climate change is now contributing to 140,000 deaths a year internationally [2], whilst another reputable source increases the estimate to 400,000 deaths a year [3]. The paper by Manfred S. Green and colleagues on climate change and health in Israel is therefore timely and important, including its focus on the nation’s adaptation policies for extreme weather events [4].

Globally, the number of reported weather-related natural disasters has more than tripled since the 1960s [2]. Green and colleagues have conducted an extensive literature review, summarising succinctly the evidence in the Israeli context on the disease and disability that can result from extreme high and low air temperatures and erratic rainfall patterns. Climate change is a major contributory factor in environmental disasters – drought, floods and landslides, storms and wildfires; and in habitat change, including loss of biodiversity, desertification, ocean acidification and sea level rise. These environmental disasters place stress upon many industries, including agriculture and fisheries. Thus, in addition to its direct health impacts, climate change also affects several key social and environmental determinants of health: clean air, safe drinking water, sufficient food and secure shelter.

Climate change both reflects and exacerbates social and health inequalities. Green and colleagues emphasise that whilst all populations will be affected by climate change, some are particularly vulnerable. Children, older people, those with low incomes and poor housing experience disproportionate effects.

### Developing resilience

The established policy response to climate change is a combination of adaptation and mitigation. In public health language, mitigation means interventions for primary prevention of severe climate change (since it is now happening and cannot be fully reversed within human timescales), whilst adaptation can be seen as secondary prevention to reduce its impact [5]. Very strong advocacy by health practitioners is needed to achieve a priority for these policies commensurate with the seriousness of the threat to human health. There are still those who deny the evidence for anthropogenic climate change despite its impressive nature [5], providing a ready excuse for inaction.

The last section of Green and colleagues’ paper makes a series of important and urgent recommendations for health policies to enable adaptation to extreme weather events. In brief, requirements for adaptation policies include undertaking baseline and regularly-updated impact and risk assessment of climate change on health; ensuring that buildings and other local infrastructure are resilient to extreme weather; ensuring an effective emergency response; and creating more green space to cool cities and absorb heavy rainfall. The WHO has recently published [6] a new tool to estimate the health and adaptation costs of climate change, to inform decisions on value-for-money.

### What’s good for the climate is good for health

Adaptation is very important, but defensive and reactive. There is a much bigger prize: many positive public health policies have the potential to reduce the greenhouse gas emissions (including carbon dioxide) that cause climate change and simultaneously to produce major health co-benefits.

The use of public transport and, particularly, active movement such as cycling and walking as alternatives to private vehicles can reduce carbon dioxide emissions and improve health by reducing obesity, cardiovascular disease, diabetes and many other conditions.

As livestock farming is the single greatest contributor to methane and carbon dioxide production [5], reducing the consumption of meat is a key policy. High levels of saturated fat intake from meat are of course implicated in cardiovascular disease and cancers. To benefit both health and the climate, it is suggested that each person eats no more than 100 grams of meat per day and has at least one meat-free day per week.

The reduction of health and social inequalities, locally, nationally and internationally, must underpin the policy response to climate change. The Climate and Health Council’s Charter (http://www.climateandhealth.org/charter) has the contraction and convergence model developed by the Global Commons Institute [7] as its central proposition: “*There is an unprecedented opportunity to reduce global health inequalities through an international agreement based on social justice, whereby national greenhouse gas emissions converge to equal per capita shares within the planet’s sustainable and finite limit. Policies to address climate change can bring greatest health gains to those with the poorest health if they are implemented with health equity and sustainability as central, linked agendas”.*

It may however be determined action to reduce greenhouse gas emissions by communities, rather than by individuals or governments, that offers the greatest hope of avoiding catastrophic consequences from climate change. The international Transition Initiatives movement includes over 150 communities promoting a community response through local food, energy, transport and cultural projects [8]. Transition Initiatives empower individuals, groups and communities: there is a rich public health literature concerning health improvement through community participation to promote both physical and mental well-being [9]. The Transition Movement in Israel should find fertile soil, because community plays such a central role in Israeli life.

In summary, Manfred S. Green and colleagues make an important contribution to public health by articulating the evidence on the health impacts of climate change and helping to frame a collective response in Israel and beyond. We encourage policymakers in Israel and elsewhere to undertake both adaptation policies to enhance resilience to adverse climate events and determined action to reduce greenhouse gas emissions.

## Competing interests

The author declares that she has no competing interests.

## Authors’ information

JG is a former Chief Executive within the National Health Service in England, and more recently an independent public health consultant. She was Moderator of the UK Public Heath Register for some years. She is a writer and activist on health and climate change, including lead editor of *The Health Practitioner’s Guide to Climate Change*, (2009, Earthscan – see references).

## Commentary on

Climate Change and Health in Israel: Adaptation Policies for Extreme Weather Events

Manfred S Green MD, PhD, Noemie Groag Pri-or MPH, Guedi Capeluto DSc, Yoram Epstein PhD, Shlomit Paz PhD

*Isr J Health Policy Res* 2013, **2:**23
